# Development of a real-time PCR assay for the identification and quantification of bovine ingredient in processed meat products

**DOI:** 10.1038/s41598-020-59010-6

**Published:** 2020-02-06

**Authors:** Xiaoyu Chen, Lixia Lu, Xiaohui Xiong, Xiong Xiong, Yuanjian Liu

**Affiliations:** 1 Coll Food Sci & Light Ind, Nanjing Tech University, Nanjing, 211816 China; 2 State Light Industry Food Quality Supervision and Detection Station, Nanjing, 211816 China

**Keywords:** DNA, Fluorescence in situ hybridization

## Abstract

In order to find fraudulent species substitution in meat products, a highly sensitive and rapid assay for meat species identification and quantification is urgently needed. In this study, species-specific primers and probes were designed from the mitochondrial *cytb* (cytochrome b) fragment for identification and quantification of bovine ingredient in commercial meat products. Bovine samples and non-bovine ones were used to identify the specificity, sensitivity, and applicability of established assay. Results showed that the primers and probes were highly specific for bovine ingredient in meat products. The absolute detection limit of the real-time PCR method was 0.025 ng DNA, and the relative detection limit was 0.002% (w/w) of positive samples. The quantitative real-time PCR assay was validated on simulated meat samples and high in the precision and accuracy. In order to demonstrate the applicability and reliability of the proposed assay in practical products, the 22 commercial meat products including salted, jerkies, and meatball were used. The results indicated the established method has a good stability in detection of bovine ingredient in real food. The established method in this study showed specificity and sensitivity in identification and quantification of beef meat in processed meat products.

## Introduction

The catalytic growth of scientific literatures related to food traceability over the last years has revealed a global issue of food adulteration^1,2^. A typical form of adulteration in meat products is the ingredient admixture or substitution of high-value species with cheaper ones to obtain lucrative profits^3^^.^ An economic loss of 45.6 million dollars has been revealed in Europe, due to the adulterated horse meat in beef products^4^. Besides the financial loss, adulterated meat products also represent a great health concern due to the unexpected exposure to food allergy^5,6^ and veterinary drug. Finally, this fraudulent conduct could also put consumers at risk of purchasing products not corresponding to their ethnical rules^7–9^.

Due to the rising living standards and rapid urbanization rates, the last years have seen a robost consumption of beef products in China. The overall beef consumption rised from 444 and 706 million kilograms in 2000, to 531 and 1982 million kilograms in 2016, for Chinese rural and urban, respectively^10^. Besides the sufficient supply, the last decades have also seen an increasing attention on beef safety and quality^11^, In particular, several recent scandals, including the horse meat scandals in Europe^12^, and the rat meat adulteration in Asia^13^ highlighted the great vulnerability of species adulteration with processed beef products. It is a challenging task to make species identification based on the residual morphological characteristics for beef products, highlighting the necessity to develop analytical assays for rapid identification of the bovine ingredient in processed meat products.

The most widely used identification method is based on DNA and a variety of techniques have been developed, including species-specific PCR (Polymerase Chain Reaction)^14–16^, PCR-RFLP (Polymerase Chain Reaction-Restriction Fragment Length Polymorphism)^9^, real-time PCR^17^, DNA barcoding^18^. Among them, a probe-based TaqMan^®^ real-time PCR is a robust assay with great rapidity and sensitivity. This technique is based on the specific hybridization of a probe, which was designed for a certain species, with the DNA in the samples to be analyzed. It has been widely used to identify meat species in commercial meat products^17,19–21^. However, there have been many problems with powerful quantitative tools for measuring meat proportion in processed foods. Accurate measurements of meat proportions were affected by different meat tissues. DNA fractions may not correlate with meat content in meat products if the mean is calibrated with DNA obtained from tissues (fatty bacon, fatless muscle, connective tissue) different to those present in the meat products^22^. To address this problem, three mixed matrices (pork, donkey and sheep with known proportions of target meat species, respectively) were prepared to be as the production of food calibrators in this study to render accurate and reproducible quantitative results.

In this study, a probe-based TaqMan^®^ real-time PCR assay for identification and quantification of bovine ingredient was developed and assessed in terms of the specificity, sensitivity and repeatability. Finally, the assay was applied on different kinds of commercial meat products.

## Materials and Methods

### Samples collection

Thirteen animal species of water buffalo (*Bubalus bubalis*), cattle (*Bos taurus*), yak (*Bos grunniens*), goat (*Capra hircus*), sheep (*Ovis aries*), horse (*Equus caballus*), donkey (*Equus asinus*), rabbit (*Oryctolagus cuniculus*), swine (*Sus scrofa*), chicken (*Gallus gallus*), duck (*Anas platyrhynchos*), goose (*Anser cygnoides*), turkey (*Meleagris gallopavo*), three fish species of grass carp (*Ctenopharyngodon idellus*), crucian (*Carassius gibelio*) and large yellow croaker (*Larimichthys crocea*), and two plant species of soybean (*Glycine max*) and corn (*Zea mays*) were purchased from local market in Nanjing, China. Visual inspection by professionals was made for species identification, followed by DNA barcoding to cross-confirm the species identity^23^ (data shown in the Supplementary Table S1). Several involved professionals include Prof. Chunbao Li, and Prof. Ming Huang, from Nanjing Agricultural University.

For method validation, 22 commercial beef products were randomly collected from local supermarket, including salted (n = 11), jerkies (n = 10), meatball (n = 1). When arrived in the laboratory, each package was labeled with an internal code, and a visual inspection of the product content was performed by morphological analysis. All specimens were stored at −20 °C for further molecular analysis.

### Binary mixture preparation

In order to avoid contamination, the surface tissue, skin, ribs, and fat layers were removed from fresh meat sample using a pair of sterile and clean scissors. The remaining tissues were ground by a high-speed crusher (XJA-100A, Specimen Mould Factory of Shanghai China), and the tissue were ground into a uniform powder from plant samples. All samples were stored at −20 °C for use.

Simulation experiment for mixed sample preparation: 100 g of ground samples (added non-toxic blue dye) were mixed by a high-speed crusher (XJA-100A, Specimen Mould Factory of Shanghai, China) for 5 min intermittently, the color of mixture was observed to be uniform, as well as the time of mixing and crushing in a high-speed crusher was determined to be 6 min intermittently.

Binary mixture preparation: Donkey tissues ground (95 g) were mixed with cattle tissues ground (5 g) using a high-speed crusher for 6 min intermittently, resulting in a 5% (w/w) sample (coded as Aa1); 80 g of donkey tissues were mixed with 20 g of Aa1 for 6 min intermittently, resulting in a 1% (w/w) sample (coded as Aa2); 50 g of donkey tissues were mixed with 50 g of Aa2, resulting in a 0.5% (w/w) sample (coded as Aa3). The 0.1% (Aa4). 0.05% (Aa5), 0.01% (Aa6), 0.005% (Aa7), 0.002% (Aa8), and 0.001% (Aa9) (w/w) samples were prepared in the same manner, and the total amount of mixture is 100 g. The sheep tissues with cattle tissues (Ab1–9), pork tissues with cattle issues (Ac1–9) and soybean tissues with cattle tissues (Ad1–9) were similarly prepared. All samples were stored at −20 °C for molecular analysis.

Moreover, in order to simulate the cooking and boiling effects that is frequently applied to industrially processed meat products^11^, the binary mixtures were also sent for autoclavation at 121 °C for 20 min.

### DNA extraction

Total DNA was extracted using the MiniBEST Universal Genomic DNA Extraction Kit (TaKaRa, Japan) for animal and fish tissue, and MiniBEST Plant Genomic DNA Extraction Kit (TaKaRa, Japan) for plants, following manufacturer’s instruction.

DNA quality and concentration were determined using a BioPhotometer D30 (Eppendorf, Hamburg, Germany) spectrophotometer and reading samples at 260 nm (A_260_) and 280 nm (A_280_). For each sample, a standard working concentration of 100 ng/µL was prepared for further analysis.

### Primer and probe design

**Figure 1:**
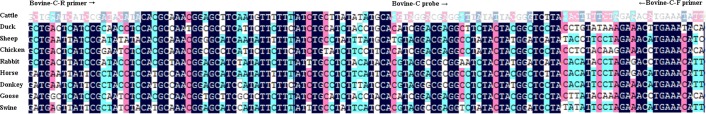
The sequence alignment of beef specific primers and probe in mitochondrial *cytb* gene against other species. Accession number of beef, duck, sheep, chicken, rabbit, horse, donkey, goose, and swine were GU249568.1, EU755252.1, AF010406.1, X52392.1, AJ001588.1, JF511458.1, JF718884.1, AY427816.1, X56295.1 respectively, target positions for designation of primers and probes were marked with closed boxes and shadow.

The mitochondrial cytochrome b (cytb) gene sequences of nine bovine and non-bovine species were downloaded from the NCBI database (https://www.ncbi.nlm.nih.gov/): cattle (GU249568.1), duck (EU755252.1), sheep (AF010406.1), chicken (X52392.1), rabbit (AJ001588.1), horse (JF511458.1), donkey (JF718884.1), goose (AY427816.1) and swine (X56295.1). All sequences were aligned using DNAMAN Multiple Alignment (Fig. 1) to find a highly conserved region. The bovine-specific primer and probe were designed by Primer express program version 3.0, and were screened for specificity to cross-species binding with other animals, fish or plant species using online BLAST tool in NCBI database (https://www.ncbi.nlm.nih.gov/Blast.cgi). The primer properties and the absence of hairpins and self-hybridization were assessed using the software Oligo calc (http://www.basic.northwestern.edu./biotools/ol igocalc.html). The designed primers and probes (Table 1) were synthesized by Sangon Biotech Corporation (Sangon Biotech, China).

**Table 1:** Oligonucleotide primer for eukaryotes and beef species.

Species	Genes	GenBank accession number	Oligonucleotides primers (5′ → 3′)	References
*Bos taurus*	Cyt b	GU249568.1	AATATTTCATGTTTCTAGAAAAGTG	This study
			GCTGAATCATCCGATACATA	
			FAM-CCCGTAATATAAGCCTCGTCCTACG-TAMRA	
Eukaryotes	18S rRNA	—	GGTAGTGACGAAAAATAACAATACAGGAC	Rojas *et al*.^24^
			ATACGCTATTGGAGCTGGAATTACC	
			FAM-AAGTGGACTCATTCCAATTACAGGGCCT-TAMRA	

The primer and probe used for positive amplification control (PAC) (Table 1) were previously reported targeting the conserved fragment in the 18S rRNA gene^24^.

### Real-time PCR protocol

The amplification by real-time PCR was accomplished in a 25 μL final volume containing 5 μL of template DNA, 12.5 μL Premix Ex Taq·Probe qPCR (TaKaRa, Japan), 0.4 μM of each primer, 0.4 μM TaqMan^®^ Probe. The reactions were performed in a Thermal Cycler system (LightCycler® 96, Roche, Switzerland) with the following program: 50 °C for 2 min and 95 °C for 10 min, followed by a 45 cycles amplification (95 °C for 15 s, and 58 °C for 1 min, annealing and extension) (recommended national standard GB/T 25165-2010 “*Protocol of identification of bovine, caprine, ovine and porcine derived materials in gelatin-Real time PCR method”*)^25^.

### Construction of calibration curve and data correction and analysis

Three mixed matrices (beef mixed with pork, donkey and sheep respectively, raw and autoclaved) were used to construct a calibration curve, (the curves were shown in the Supplementary Figs. S1–S3) The results indicate that it is available for the mixed matrices (beef with pork, autoclaved) to be as production of food calibrators in this study, and that the results agree with the ENGL criteria (the method could be accepted that the R^2^ value should be above 0.98, the slope of standard curve was between 3.1 and 3.6, and the PCR efficiency should range between 90% and 110%^26^).

To apply the quantitative assay to heavily-processed meat products, PCR data were normalized with threshold cycle (Cq) values received by the bovine-specific system and eukaryotic system to construct a calibration curve^17^. The calibration curve was created with reference mixtures containing known beef content (0.01, 0.05, 0.1, 0.5, 1, 5, 100%,w/w) in heated pork.

As historically reported by Fajardo^27^, the Cq values received from the blind sample using the bos-specific system (CqB) were determined by Eq. (1)

1 $${\rm{CqS}}={\rm{CqEU}}\ast {\rm{CqB}}/{\rm{CqEUB}}$$

Where CqS is the normalized Cq value of the sample in the Eq. (1), CqEU is the average Cq value of the binary model mixtures with the endogenous PCR system, and CqEUB is the Cq value of the sample detected with the endogenous PCR system^27^.

The target species in blind samples can be estimated by linear interpolation with the calibration curve of CqS values generated from samples. The correlation between CqS values and content (C) is defined by Eq. (2)

2 $${\rm{CqS}}={\rm{a}}\,{\rm{LogC}}+{\rm{b}}$$

Where a is the slope and b is the intercept in the Eq. (2).

### Limit of quantifification (LOQ)

The LOQ is the lowest amount of analyte in a sample that can be reliably quantified at an acceptable level of precision, accuracy, and repeatability^28^. Besides, the LOQ should be ≤ the minimum value included in the dynamic range, and its assessment should be obtained from a abundant number of detection data, at least 15, by analogy with the requirement set for the estimates of Relative Repeatability Standard Deviation (RSDr) (RSDr should be ≤25% over the whole dynamic range of the PCR modules individually)^26^.

### Compliance with ethical standards

Ethical Approval this article does not contain any studies with human participants or animals performed by any of the authors.

## Results and Discussion

### Sequence analysis and primer design

The primer and probe design is a critical step in TaqMan probe assay and it must contain adequate intra-species conserved sequences and inter-species polymorphism^29^. Several sequences of mitochondrial and nuclear genes were aligned using DNAMAN Multiple Alignment to obtain a short-length and intra-species conserved and inter-species fragment. Finally, a short fragment on mitochondrial *cytb* region was determined to meet the requirements.

Following absolute intra-species conservation (the 3′ sequences of primer and the whole sequences of probe), the bovine-specific primer and probe were designed by Primer express program version 3.0, and screened for specificity to cross-species binding with other animals, fish or plant species using online BLAST tool in NCBI database (https://www.ncbi.nlm.nih.gov/Blast.cgi). The amplified target sequence is short-length of 119 bp in mitochondrial *cytb* region, since the short-length nucleic acid sequences are extraordinarily stable under harsh conditions and mitochondrial genes are present in multiple copies^1,30^.

### Specificity test

**Figure 2:**
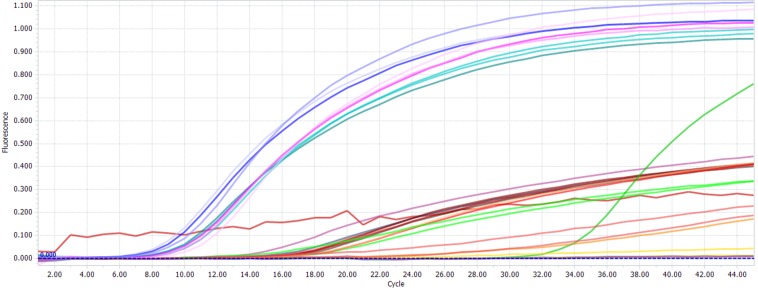
The real-time PCR of specific amplification curve.

Exclusivity was 100% (false positive rate 0%) for all employed primer and probe systems while tested the plant species, with no cross reactivity observed. The Table 2 (the real-time PCR of specific amplification curve are shown in Fig. 2) showed that the Cq values of bovine (cattle, water buffalo and yak) as follow: 9.00 ± 0.02, 9.20 ± 0.01, 9.60 ± 0.02, respectively, were considered as the positive amplification, whereas there was no amplification achieved with DNA from ten non-target animal species and two plant species. The eukaryotic system was used as positive amplification control (PAC) to avoid false negative amplification, and positive amplification by enkaryotic system was obtained for all species (Table 2).

**Table 2:** Specificity of the real-time PCR system.

Common name	*Scientific name*	Bs.S.S	P.A.C^b^
Cattle	*Bos taurus*	9.00 ± 0.02^a^	14.70 ± 0.18
Water buffalo	*Bubalus bubalis*	9.20 ± 0.01^a^	15.03 ± 0.08
Yak	*Bos grunniens*	9.60 ± 0.02^a^	15.24 ± 0.03
Sheep	*Ovis aries*	Nat	20.10 ± 0.03
Goat	*Capra hircus*	Nat	19.30 ± 0.13
Swine	*Sus scrofa*	Nat	12.92 ± 0.02
Horse	*Equus caballus*	Nat	14.90 ± 0.02
Donkey	*Equus asinus*	Nat	13.00 ± 0.04
Rabbit	*Otyctolagus cuniculus*	Nat	16.40 ± 0.02
Chicken	*Gallus gallus*	Nat	18.90 ± 0.08
Goose	*Anser cygnoides*	Nat	17.84 ± 0.16
Duck	*Anas platyrhynchos*	Nat	16.00 ± 0.05
Turkey	*Meleagris gallopavo*	Nat	17.61 ± 0.03
Grass Carp	*Ctenopharyngodon idellus*	Nat	12.90 ± 0.14
Crucian	*Carassius gibelio*	Nat	11.80 ± 0.05
Large yellow croaker	*Larimichthys crocea*	Nat	14.20 ± 0.04
Corn	*Zea mays*	Nat	15.90 ± 0.10
Soybean	*Glycine max*	Nat	18.40 ± 0.11

^a^Average Cq value ± SD shown from triplicate PCR reaction from each template.

Nat indicates no positive signal before 32 PCR cycles.

Bs.S.S bovine-specific system on the Cyt b gene.

^b^Positive amplification control on the 18S rRNA gene.

### Sensitivity of the method

Considering the European Network of Genetically Modified Organism (GMO) Laboratories guidelines^26^, the limit of detection (LOD) of the real-time PCR assay was determined using serially diluted DNA from autoclaved beef to be as a calibrator. The LOD is when there are 26 or more positive amplification times out of 27 reactions, according to the ≥95% confidence level rule.

PCR amplification of DNA from binary model mixtures were used to evaluate the relative LOD. The run was performed under repeatable conditions for reliable generation of absolute and relative LOD^28^.

**Figure 3:**
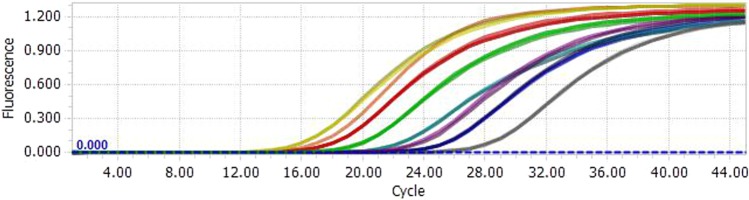
The real-time PCR data of amplification curves obtained directly with the instrument. (For serially diluted DNA).

For the absolute LOD test (Table 3, Cq values and respective SD were shown in the Supplementary Table S2), results showed that positive signals appeared 26 or more out of 27 reactions from 0.025 ng cattle meat DNA. In conclusion, detected the lowest level was 0.025 ng of DNA at a 95% confidence level. (The real-time PCR data of amplification curves obtained directly with the instrument for serially diluted DNA are shown in Fig. 3)

**Table 3:** Absolute LOD values from real-time PCR for cattle DNA.

Sample species	Absolute amount of DNA (ng)										
	matrix	1000	50	10	5	1.0	0.5	0.10	0.05	0.025	0.010
*Bos taurus*	donkey	27/27	27/27	27/27	27/27	27/27	27/27	27/27	27/27	26/27	21/27 ^C^
	sheep	27/27	27/27	27/27	27/27	27/27	27/27	27/27	27/27	27/27	26/27
	pork	27/27	27/27	27/27	27/27	27/27	27/27	27/27	27/27	27/27	26/27
	horse	27/27	27/27	27/27	27/27	27/27	27/27	27/27	27/27	27/27	22/27
	rabbit	27/27	27/27	27/27	27/27	27/27	27/27	27/27	27/27	26/27	26/27
	chicken	27/27	27/27	27/27	27/27	27/27	27/27	27/27	27/27	27/27	27/27
	duck	27/27	27/27	27/27	27/27	27/27	27/27	27/27	27/27	26/27	25/27
	goose	27/27	27/27	27/27	27/27	27/27	27/27	27/27	27/27	26/27	26/27
	grass carp	27/27	27/27	27/27	27/27	27/27	27/27	27/27	27/27	27/27	24/27
	soybean	27/27	27/27	27/27	27/27	27/27	27/27	27/27	27/27	27/27	25/27
	corn	27/27	27/27	27/27	27/27	27/27	27/27	27/27	27/27	27/27	26/27

^c^The species was detected 21 out of 27 times.

**Figure 4:**
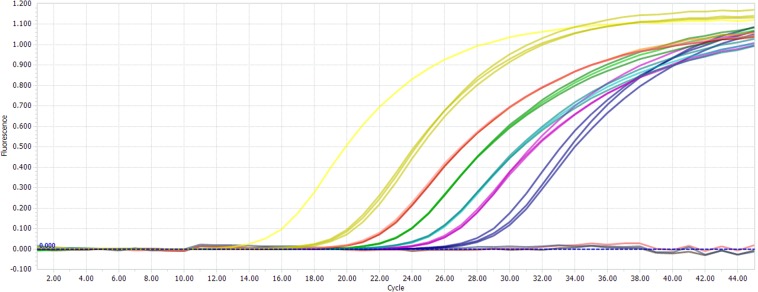
The real-time PCR data of amplification curves obtained directly with the instrument. (For serially diluted mixtures).

For the relative LOD test (Table 4, Cq values and respective SD were shown in the Supplementary Table S3), the positive amplification appeared in 26 or more out of 27 reactions except 0.001% (w/w) cattle meat. In conclusion, the lowest cattle meat content could be test was 0.002% (w/w) at a 95% confidence level. Similar result has also been reported in previous study which concluded that cooking and sterilization processed do not affect significantly the response of PCR to different concentrations of DNA, as well as non-target DNA in the meat admixtures do not affect the PCR efficiency^22^. (The real-time PCR data of amplification curves obtained directly with the instrument for serially diluted mixtures are shown in Fig. 4)

**Table 4:** Relative LOD values from real-time PCR for cattle DNA.

Sample species	Percentage (w/w)										
	matrix	code	5%	1%	0.5	0.1	0.05	0.01	0.005	0.002	0.001
*Bos taurus* (raw)	donkey	Aa1–9^d^	27/27	27/27	27/27	27/27	27/27	27/27	27/27	27/27	26/27
	sheep	Ab1–9	27/27	27/27	27/27	27/27	27/27	27/27	27/27	27/27	25/27 ^e^
	pork	Ac1–9	27/27	27/27	27/27	27/27	27/27	27/27	27/27	26/27	24/27
	soybean	Ad1–9	27/27	27/27	27/27	27/27	27/27	27/27	27/27	27/27	27/27
	donkey	Aa1–9	27/27	27/27	27/27	27/27	27/27	27/27	27/27	26/27	26/27
*Bos taurus* (autoclaved)	sheep	Ab1–9	27/27	27/27	27/27	27/27	27/27	27/27	27/27	26/27	15/27
	pork	Ac1–9	27/27	27/27	27/27	27/27	27/27	27/27	27/27	27/27	17/27
	soybean	Ad1–9	27/27	27/27	27/27	27/27	27/27	27/27	27/27	26/27	20/27

^d^Number 1,2,3,4,5, 6,7, 8, and 9 represent samples of 5%, 1%, 0.5%, 0.1%, 0.05%, 0.01%, 0.005%, 0.002%, 0.001%, respectively.

^e^The species was detected 25 out of 27 times.

The LOD was determined to be 0.005 ng/μL pure bovine DNA, which is equivalent to 0.025 ng DNA. The relative sensitivity of the real-time PCR assay using the reference mixtures was 0.002% (w/w). While previous studies reported an absolute LOD of 0.1 ng using multiplex PCR for horse, dog and pork^31^ and 0.01 ng using real time PCR for horse^8,32^. (Safdar and Junejo 2016)^14^ reported similar analytical sensitivities, namely 0.01% sensitivity for horse, soybean, sheep, poultry, pork, and cow, respectively. Therefore, the detection limit of the real-time PCR assay established in this study was equal or lower to previously reported values.

### Linearity and calibration curve on heat-treated samples

Previous study obtained the conclusion that the cooking and sterilization used do not affect significantly the response of PCR to different concentrations of DNA, and non-target DNA in the admixtures do not affect the PCR efficiency^22^. López-Andreo study showed^33^ that there is a linear correlation of Cq values and Log DNA found by testing single-species or mixed DNA purified from raw meat using the Taqman^®^ MGB detectors of *bos* and *sus*, with a PCR efficiency approaching doubling each cycle. Kim *et al*. normalized Cq values utilizing species-specific PCR system and the endogenous PCR system to remove errors from the complex food matrix and processing treatments as well as construct the calibration curve, yielded a linear correlation of Cq values and Log (pork meat contents)^17^.

**Figure 5:**
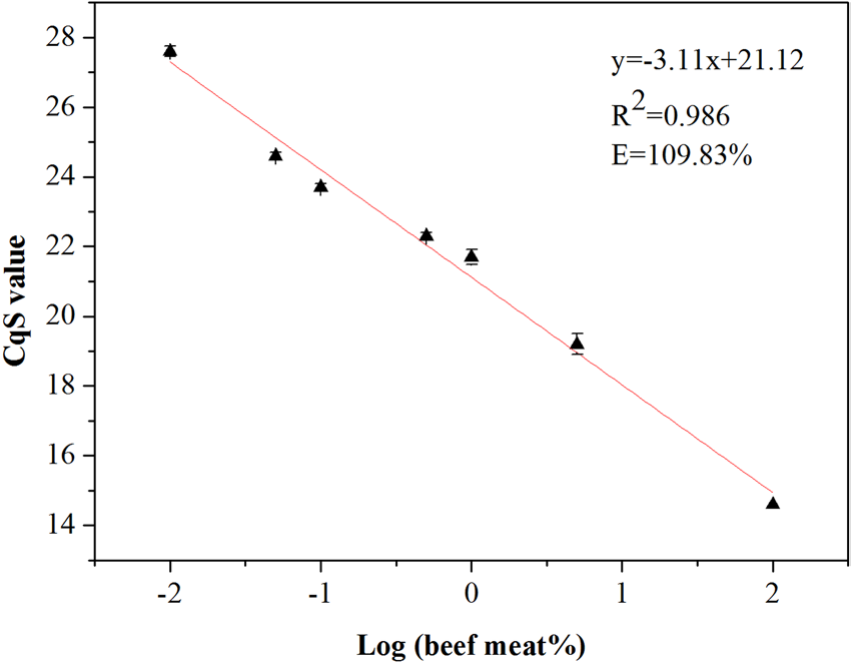
Normalized calibration curve for the reference mixture with the average Cq values of reference mixtures using the endogenous PCR system (n = 7).

Selected the Taqman^®^ FAM detectors bovine, the calibration curve was created by plotting the normalized Cq values and the logarithm of autoclaved beef contents in this study(section 2.6).The calibration curve was obtained from repeated data on three different weeks and mean Cq values and the normalized Cq values were shown in Table 5. The Fig. 5 showed that the correlation coefficient (R^2^) of calibration curve was 0.986, the correlation slope was −3.11 ± 0.006, and the PCR efficiency was 109.83% (The PCR efficiency, which was calculated using the equation E = [10^−1/slope^−1 (3)], other relevant results are shown in Table 6). According to the ENGL criteria, the method could be accepted that the R^2^ value should be above 0.98, the slope of standard curve was between 3.1 and 3.6, and the PCR efficiency should range between 90% and 110%^26^.The GMO criteria were used to estimate PCR efficiency in this study because there is no appropriate quantification criterion for detecting of meat products at present^17^. The qPCR (Quantitative PCR) assay constructed in this study meets above criteria, and showed good linearity and PCR reaction efficiency, thus was regarded as a available tool of quantify beef content. The study data showed that there were no significant difference on the standard curve of R^2^ values and slopes using raw and autoclaved reference mixture meat.

**Table 5:** The values of normalized Cq (a) and mean (b) obtained from quantitative PCR using pork-specific and eukaryotic systems to create the calibration curve.

	Log (beef meat %)	CqB	CqEUB	CqEU	CqS
**(a)**					
Week 1	2.0	14.5	19.7	19.8	14.6
	0.7	19.0	19.9	19.8	18.9
	0.0	21.8	20.0	19.8	21.5
	−0.3	22.2	19.8	19.8	22.2
	−1.0	23.5	19.7	19.8	23.6
	−1.3	24.4	19.7	19.8	24.5
	−2.0	27.5	19.7	19.8	27.6
Week 2	2.0	14.5	19.7	19.8	14.6
	0.7	19.2	19.9	19.8	19.1
	0.0	22.1	20.0	19.8	21.9
	−0.3	22.6	20.0	19.8	22.4
	−1.0	23.5	19.6	19.8	23.7
	−1.3	24.5	19.7	19.8	24.6
	−2.0	27.5	19.8	19.8	27.5
Week 3	2.0	14.7	20.2	20.0	14.6
	0.7	19.5	20.0	20.0	19.5
	0.0	22.1	20.2	20.0	21.8
	−0.3	22.6	20.2	20.0	22.4
	−1.0	23.5	19.8	20.0	23.8
	−1.3	24.3	19.7	20.0	24.7
	−2.0	27.6	19.9	20.0	27.8
**(b)**					
**Log (beef meat %)**	**Normalized Ct value (CqS)**				
	**Week 1**	**Week 2**	**Week 3**	**Mean**	**SD**
2.0	14.6	14.6	14.6	14.6	0.0
0.7	18.9	19.1	19.5	19.2	0.3
0.0	21.5	21.9	21.8	21.7	0.2
−0.3	22.2	22.4	22.4	22.3	0.1
−1.0	23.6	23.7	23.8	23.7	0.1
−1.3	24.5	24.6	24.7	24.6	0.1
−2.0	27.6	27.5	27.8	27.6	0.2

**Table 6:** The raw data of slope, efficiency, and R^2^ obtained from quantitative PCR.

	Slope	Efficiency (%)	R^2^
Week 1	−3.11	109.65	0.988
Week 2	−3.10	110.18	0.984
Week 3	−3.11	109.65	0.984
Mean	−3.11	109.83	0.986

The Linear dynamic range over which a reaction is linear (the maximun to the minimun quantifiable amount created by means of a calibration curve)^34^. The results incidated that the dynamic range is between 0.01% and 100% (w/w) in this study.

### Precision, accuracy and trueness

Specific group of food matrix-cattle samples (at five different percentages) were prepared. Cattle tissues (75 g) were mixed with 25 g of pork tissues using a high-speed crusher for 6 min, resulting in a 75% (w/w) sample (coded as Ba1). 80 g of Ba1 were mixed with 40 g of pork tissues for 6 min, resulting in a 50% (w/w) sample (coded as Ba2). 50 g of Ba2 were mixed with 50 g of pork tissues for 6 min, resulting in a 25% (w/w) sample (coded as Ba3). 20 g of Ba3 were mixed with 80 g of pork tissue for 6 min, resulting in a 5% sample (coded as Ba4). The pure cattle tissue was coded as Ba5 (100%,w/w). The mixtures were sent for autoclavation at 121 °C for 20 min. The DNA was extracted from mixtures above, and store at −20 °C for further analysis.

In order to assess the precision (relative standard deviation, RSD) and accuracy (relative mean deviation) of the method, various meat fractions with defined proportions of beef, covering the dynamic range of the assay, were analyzed under repeatability conditions. The results indicated (Table 7) that the developed assay has a good performance, with the precision, accuracy and trueness of the method lying well the acceptance criterion of ≤25%^26^.

**Table 7:** The calculated precision, accuracy trueness of the method as compiled from 3 independent measurements. The results are compiled from 3 independent runs with an average 18 measurement point.

Actual beef proportion (%)	Measured beef proportion (%)	Precision (%)	Accuracy (%)	Trueness (%)
100	100.60	0.04	3.13	0.60
75	72.53	0.07	3.76	3.38
50	48.51	0.01	0.35	3.08
25	24.21	0.07	0.96	3.29
5	4.20	0.05	0.07	19.05

### Application of method on a reference gene for beef meat species quantification in commercial meat products

At present, many methods for identification of meat species have been published. While the majority of these methods are of a qualitative nature, more recent scandals in the food industry require sensitive quantitative methods to distinguish more accurately between deliberate adulteration and inadvertent contamination^8^. In this study, 22 commercial processed meat products were selected from different manufacture processes, which could affect DNA degradation and complex food matrices. The quantitative analysis of the meat products was carried out on three different weeks, the results (Table 8) were obtained from repeated data.

**Table 8:** Results of Real-time PCR assay performed on commercial meat products.

No.	Product styles	Labeled as	Cq values			Estimated pork meat contents (%)		
			Mean	SD	RSD	Mean	SD	RSD
P-1	salted	Bovine	17.39	0.04	0.23	15.83	0.47	2.96
P-2	salted	Bovine	15.57	0.06	0.38	60.78	2.61	4.29
P-3	salted	Bovine	17.46	0.09	0.46	15.09	0.91	6.02
P-4	salted	Bovine	16.12	0.09	0.53	40.48	2.61	6.44
P-5	salted	Bovine	15.84	0.57	3.64	53.33	8.09	15.16
P-6	salted	Bovine	15.08	0.13	0.83	87.99	8.15	9.26
P-7	salted	Bovine	15.17	0.16	1.03	82.24	9.16	11.13
P-8	salted	Bovine	14.94	0.06	0.43	96.72	0.05	0.06
P-9	salted	Bovine	14.80	0.02	0.14	107.69	1.59	1.48
P-10	salted	Yak	16.63	0.21	1.26	27.80	4.40	1.58
P-11	salted	Bovine	16.51	0.13	0.79	30.45	2.93	9.61
J-1	jerky	Bovine	14.68	0.09	0.63	117.85	8.07	6.85
J-2	jerky	Bovine	16.49	0.21	1.27	31.04	4.58	14.70
J-3	jerky	Bovine	18.89	0.03	0.13	5.20	0.10	1.86
J-4	jerky	Bovine + Fish	16.19	0.18	1.17	38.73	5.29	13.67
J-5	jerky	Bovine + Pork	16.73	0.10	0.60	26.05	2.23	8.58
J-6	jerky	Bovine	14.82	0.07	0.48	106.17	5.56	5.23
J-7	jerky	Bovine	18.69	0.02	0.08	6.06	0.07	1.13
J-8	jerky	Bovine	14.86	0.05	0.36	103.0	4.58	4.44
J-9	jerky	Bovine	17.21	0.08	0.45	18.10	1.03	5.68
J-10	jerky	Bovine	18.04	0.16	0.89	9.83	1.16	11.76
M-1	meatball	Bovine + Chicken + Duck	16.84	0.15	0.89	23.92	2.39	10.00

In this work, positive amplification control (PAC) and negative amplification control (NAC) were set up, all PAC appeared amplification signals, and all NAC is no amplification signal.

Extraction DNA concentration from all samples were within the 10^2^ range.

There are differences of beef meat contents between these detected by the assay and on the product labels (Table 8), as the processing degree affects DNA degradation result in differences of data tested from different types of commercial processed meat products. As for salted samples as follow 1, 3, 4,10 and 11, the detected beef meat contents are far lower than the contents on labels (Table 8). It is a reasonable explanation that high salt content affects DNA extraction efficiency and PCR amplification, or it might be possibly that there is serious adulteration during processing of meat products. And the beef meat contents estimated for the other salted samples are consistent with the ones given on the label. The beef meat contents for jerky samples (2, 3, 7, 9 and 10) are quantified between 5.20% and 38.73% (Table 8), which are extremely lower than the other ones. The detected beef meat content of meatball for sample 1 is 23.92% (as shown in Table 8).

Due to the diversity and the easier changes of composition of meat products during processing, the limit of relative quantitative method of fluorescence PCR for precision lies in standard products, copy number ratio and mass content conversion. Some studies indeed show that the accuracy of real-time PCR assays is significantly increased while calibration sausages on commercial meat products are similar to the detected meat samples, are employed^12^. Besides, the challenge lies in the production of calibration sausages that can contain the various commercial meat products. Clearly, it is difficult on an analytical viewpoint, where food control laboratories^8^. Based on the previous studies for detection of a single type of processed meat product, this study simulated commercial meat product matrixes and developed the PCR assay, which can be applied to various common types of processed meat products.

## Conclusion

The assay developed in this study offers a reliable quantification strategy for beef meat products, which was successfully extended to different type beef meat products with varying matrix and composition. The real-time PCR method developed in this work can be used for identification and quantification of bovine ingredient in commercial processed meat products.

## Supplementary information

Supplementary information

## Data Availability

All data generated or analysed during this study are included in this published article (and its Supplementary information files).
