# Supplementary material for: Development of a real-time PCR assay for the identification and quantification of bovine ingredient in processed meat products
Source: Sci Rep. 2020 Feb 6;10:2052. doi: 10.1038/s41598-020-59010-6 (PMC7004997; doi:10.1038/s41598-020-59010-6)

**Development of a real-time PCR assay for the identification and quantification of bovine ingredient in processed meat products**

Xiaoyu Chen^1^·Lixia Lu*^1,2^·Xiaohui Xiong^1,2^·Xiong Xiong^1^·Yuanjian Liu^1^

^1^ Coll Food Sci & Light Ind, Nanjing Tech University, Nanjing, 211816, China

^2^ State Light Industry Food Quality Supervision and Detection Station, Nanjing, 211816, China

Lixia Lu* (Corresponding Author）

E-mail address: llxhn66@126.com

Table S1 The results of PCR sequencing

| Labeledname | Sequence (5’→3’) | Identification results |
| --- | --- | --- |
| *Bubalus bubalis* | TAAGTCAGTATCTGATAATACCCTGACCATTAACAGCTAATAAAAATAACCCAGTAATAAATAATTTATTTATTACACTGTTAATCCACACAGGAGTGCACCAAGGAAAGATTAAAAGAAGTAAAAGGAACTCGGCAAACACAAACCCCGCCTGTTTACCAAAAACATCACCTCCAAGCATCCCTAGTATTGGAGGCACTGCCTGCCCAGTGACAACCGTTAAACGGCCGCGGTATCCTGACCGTGCAAAGGTAGCATAATCACTTGTTCTTTAAATAGGGACTTGTATGAATGGCCACACGAGGGTTTTACTGTCTCTTACTTCCAATCAGTGAAATTGACCTTCCCGTGAAGAGGCGGGAATGCACTAATAAGACGAGAAGACCCTATGGAGCTTTAACTAATCAGCCCAAAGAAAAATAAACCAAACCGCTAAGGAGTAACAACACTCTTCATGAGCTGACAGTTTCGGTTGGGGTGACCTCGGAGAATAAAAAATCCTCCGAGCGATTTTAAAGACTAGACCTACAAGTCAAATCGCTCTATCGCTCATTGATCCAAAAATTTTGATCAACGGAACAAGTTACCCTAGGGATAACAGCGCAATCCTATTCAAGAGTCCATATCGACAATAGGGTTTACG | *Bubalus bubalis* |
| *Bos taurus* | TTTATCTACTATTTGGTGCTTGGGCCGGTATAGTAGGAACAGCTCTAAGCCTTCTAATTCGCGCTGAATTAGGCCAACCCGGAACTCTGCTCGGAGACGACCAAATCTACAACGTAGTTGTAACCGCACACGCATTTGTAATAATCTTCTTCATAGTAATACCAATCATAATTGGAGGATTCGGTAACTGACTTGTTCCCCTAATAATTGGTGCTCCCGATATAGCATTTCCCCGAATAAATAATATAAGCTTCTGACTCCTCCCTCCCTCATTCCTACTACTCCTCGCATCCTCTATAGTTGAAGCTGGGGCAGGAACAGGCTGAACCGTGTACCCTCCCTTAGCAGGCAACCTAGCCCATGCAGGAGCTTCAGTAGATCTAACCATTTTCTCTTTACACTTAGCAGGAGTTTCCTCAATTTTAGGAGCCATCAACTTCATTACAACAATTATCAACATAAAGCCCCCCGCAATGTCACAATACCAAACCCCTCTGTTCGTATGATCCGTAATAATTACCGCCGTACTACTACTACTCTCGCTCCCTGTATTAGCAGCCGGCATCACAATGCTATTAACAGACCGGAACCTAAATACAACCTTCTTCGACCCGGCAGGAGGAGGAGACCCTATTCTATTCAACAC | *Bos taurus* |
| *Bos grunniens* | TTTAACTCTATTTGGAGCCTGGGCCGGTATAGTAGGAACAGCTCTAAGCCTTCTAATTCGCGCTGAATTAGGCCAACCCGGAACCCTGCTCGGAGACGACCAAATCTACAACGTAGTTGTAACCGCACACGCATTTGTAATAATCTTCTTTATAGTAATGCCAATTATAATTGGAGGATTCGGTAACTGACTTGTTCCTCTAATAATTGGCGCTCCCGATATGGCATTCCCCCGAATAAACAACATAAGCTTCTGACTCCTCCCTCCCTCATTTCTACTGCTCCTCGCATCCTCTATAGTTGAAGCTGGAGCAGGAACAGGCTGAACCGTGTACCCTCCCTTAGCAGGCAACCTAGCCCATGCAGGAGCCTCAGTAGATCTAACCATCTTCTCTTTACACTTAGCAGGAGTTTCCTCTATTTTAGGAGCCATCAACTTCATTACAACAATTATCAACATAAAGCCCCCCGCAATGTCACAGTACCAAACTCCCCTATTCGTATGATCCGTAATAATCACCGCCGTACTATTACTCCTCTCACTCCCTGTACTAGCAGCCGGCATTACAATGCTGCTAACAGACCGGAACCTAAATACAACCTTCTTCGACCCAGCAGGAGGAGGAGACCCCATTTTATACCAA | *Bos grunniens* |
| *Capra hircus* | AATATAAATAAATTTTACTGTATTTTTAAAAGTTAGTCTAAAAAGGTTCAGCCTTTTAGAAATGGATACAACCTTCACTAGAGAGTAAGACTTTACAACACCATAGTAGGCCTAAAAGCAGCCATCAATTAAGAAAGCGTTAAAGCTCAACAATAAAAATAAAATTAATCCCAACAATAGTACAACTAACTCCTAGACCTAATACTGGACCACTCTATTATTAAATAGAAGCAATAATGTTAATATGAGTAACAAGAAATATTTTCTCCCTGCACAAGTTTAAGTCAGTATCTGATAATATTCTGACTGTTAACAGTAAATAAAAACAACCTAACGATAAATAATTTATTAATTACACTGTTAACCCAACACAGGAGTGCACCCAGGAAAGATTAAAAGAAGTAAAAGGAACTCGGCAAACACAAACCCCGCCTGTTTACCAAAAACATCACCTCCAGCATTTCCAGTATTGGAGGCACTGCCTGCCCAGTGACTAAACGTTAAACGGCCGCGGTATTCTGACCGTGCAAAGGTAGCATAATCATTTGTTCTCTAAATAAGGACTTGTATGAACGGCCACACGAGGGTTTTACTGTCTCTTACTTCCAATCAGTGAAATTGACCTCCCCGTGAAGAGGCGGGAATG | *Capra hircus* |
| *Ovis aries* | CTTCTTTTGGTGCCTGAGCTGGTATAGTAGGAACCGCCTTAAGCCTACTAATTCGCGCCGAACTAGGCCAACCCGGAACTCTACTCGGAGATGACCAAATCTACAACGTAATTGTAACCGCACATGCATTTGTAATAATTTTCTTTATAGTAATGCCTATTATAATCGGTGGATTTGGCAACTGACTAGTTCCTCTGATAATTGGAGCCCCTGATATAGCATTTCCTCGGATAAATAACATAAGCTTTTGACTTCTTCCCCCATCTTTCCTGTTACTCCTAGCATCCTCTATGGTTGAGGCCGGAGCAGGAACAGGTTGAACCGTATACCCTCCTCTAGCAGGCAACCTAGCCCATGCAGGAGCCTCAGTAGATCTAACTATTTTCTCCCTACACCTGGCAGGTGTCTCTTCAATTCTAGGAGCCATTAATTTTATTACAACTATTATTAATATAAAACCCCCTGCGATGTCACAGTATCAAACCCCCTTGTTTGTATGATCTGTACTAATTACTGCCGTACTTCTCCTTCTCTCACTTCCTGTATTAGCAGCTGGTATCACAATACTACTAACGGACCGAAACCTGAATACAACCTTTTTTGACCCAGCAGGAGGAGGAGACCCTATCCTATATCAACACC | *Ovis aries* |
| *Equus caballus* | GTACCTCCTATTCCGCGCTTGAGCTGGAATAGTAGGAACTGCCCTAAGCCTCCTAATCCGTGCTGAATTAGGCCAACCTGGGACCCTACTAGGAGATGATCAGATCTACAATGTTATTGTAACCGCCCATGCATTCGTAATAATTTTCTATATGGTCATACCCATTATAATCGGAGGATTCGGAAACTGATTAGTCCCCCTGATAATTGGAGCACCTGTTATAGCTTTCCCCCGAATAAACAACATAAGCTTCTGATTACTTCCCCCATCATTCCTACTTCTTCTCGCTTCCTCAATAATTGAAGCAGGTGCCGGAACAGGCTGAACCGTATATCCTCCTCTAGCTGGAAATCTGGCGCATGCAGGAGCCTCTGTTGACTTAACCATTTTCTCTCTCCACCTGGCTGGGGTGTCCTCGATTTTAGGTGCCATCAACTTTATTACCACAATCATTAACATAAAACCACCAGCTCTATCCCAATATCAAACCCCCCTATTCGTTTGATCTGTCGTTATTACGGCAGTACTCCTTCTCCTAGCCCTCCCGGTCCTAGCAGCAGGCATTACCATGCTTCTCACAGACCGTAACCTAAACACTACTTTCTTCGACCCCGCAGGAGGAGGGGATCCAATCCTTTATCAACACG | *Equus caballus* |
| *Equus asinus* | CTCTGTAGCTCTATTTCGGCGCTTGAGCTGGAATAGTAGGAACCGCCCTAAGCCTCCTAATCCGTGCTGAATTAGGTCAACCTGGGACCCTGCTGGGAGATGATCAGATCTACAATGTTATTGTAACTGCCCATGCATTCGTAATAATCTTCTTCATAGTCATACCCATCATGATCGGAGGATTTGGGAACTGATTAGTTCCCTTAATAATTGGAGCACCCGATATAGCCTTTCCCCGAATAAACAACATAAGCTTCTGATTACTTCCCCCATCATTCCTACTTCTTCTTGCTTCCTCAATAATTGAAGCAGGCGCTGGAACAGGCTGAACCGTATATCCTCGCCTAGCTGGAAATCTAGCGCACGCAGGGGCTTCTGTTGACTTAACCATCTTCTCCCTTCACCTAGCTGGTGTATCTTCAATTTTAGGTGCCATCAATTTCATTACCACAATCATCAACATAAAACCACCAGCCCTGTCCCAGTATCAAACCCCTCTATTCGTTTGATCCGTCCTCATTACGGCAGTACTCCTTCTCCTAGCTCTTCCAGTCCTAGCAGCAGGTATTATTATGCTTCTCACAGACCGTAACTTAAACACCACCTTCTTGG | *Equus asinus* |
| *Oryctolagus cuniculus* | GGCATGGTGGCAACAGCCCTCAGCCTGCTAATTCGAGCAGAATTAGGTCAGCCAGGGACTCTACTCGGGGATGATCAAATCTATAATGTAATCGTCACCGCACATGCCTTTGTAATAATCTTCTTTATAGTCATGCCTATTATAATTAGAGGCTTCGGGAACTGGCTTGTCCCCCTGATAATTGGGGCTCCTGACATAGCCTTCCCCCGAATAAATAATATGAGCTTCTGACTTCTCCCCCCTTCATTCCTTCTTCTACTAGCCTCCTCAATAGTAGAAGCTGGGGCGGGGACTGGCTGAACTGTTTATCCACCTCTAGCCGGTAATCTTGCACATGCTGGAGCCTCAGTGGATCTTACTATTTTCTCCCTTCACTTAGCTGGAGTATCATCTATTTTAGGGGCTATTAACTTTATTACAACTATTATTAATATGAAACCCCCTGCAATATCTCAATATCAAACCCCCTTATTCGTATGATCTGTTCTAATCACAGCCGTACTTCTTCTTCTCTCTTTACCAGTCCTAGCTGCTGGCATTACAATGCTTTTAACAGACCGAAACTTAAATACAACCTTCTATGATCCTGCAGGAGGAGGAGACCCTATCCTCTACCAACACCTATTCTGATTCTTTGGCCACCGA | *Oryctolagus cuniculus* |
| *Sus scrofa* | CTACTATTTGGTGCCTGAGCAGGAATAGTGGGCACTGCCTTGAGCCTACTAATTCGCGCTGAACTAGGTCAGCCCGGAACCCTACTTGGCGATGATCAAATCTATAATGTAATTGTTACAGCTCATGCCTTTGTAATAATCTTCTTTATAGTAATACCCATTATGATTGGAGGTTTTGGTAACTGACTCGTACCGCTAATAATCGGAGCTCCCGATATGGCCTTTCCACGTATAAACAACATAAGTTTCTGACTACTTCCACCATCCTTCCTATTACTTCTGGCATCCTCAATAGTAGAAGCCGGAGCGGGTACTGGATGAACTGTATACCCACCTTTAGCTGGAAACTTAGCCCATGCAGGGGCTTCAGTTGATTTAACAATTTTCTCCCTACACCTTGCAGGTGTATCATCAATCCTAGGGGCTATTAATTTCATTACCACAATTATTAACATAAAACCCCCCGCAATGTCTCAATACCAAACACCCCTGTTTGTCTGATCAGTACTAATCACAGCCGTACTACTTCTACTATCCCTGCCAGTTCTAGCAGCTGGCATTACTATACTACTGACAGACCGCAACCTGAACACAACCTTTTTTGATCCAGCAGGTGGTGGAGACCCTATCCTTTATCAACACTTG | *Sus scrofa* |
| *Gallus gallus* | GGCACATGGGCGGGCATAGCCGGCACAGCACTTAGCCTTCTAATTCGCGCAGAACTAGGACAGCCCGGAACTCTCTTAGGAGACGATCAAATTTACAATGTAATCGTCACAGCCCATGCTTTCGTCATAATCTTCTTTATAGTTATACCCATCATGATCGGTGGCTTCGGAAACTGACTAGTCCCACTTATAATCGGTGCCCCAGACATAGCATTCCCCCGCATAAATAACATAAGCTTCTGACTCCTCCCTCCCTCCTTCCTTCTCCTACTAGCCTCATCTACCGTAGAAGCTGGGGCCGGCACAGGATGGACAGTTTACCCCCCTTTAGCCGGCAACCTAGCCCACGCTGGCGCATCAGTAGACCTAGCCATCTTTTCATTACACTTAGCAGGTGTTTCCTCCATTCTAGGAGCCATCAACTTTATCACTACCATCATCAACATAAAACCCCCCGCACTGTCACAATACCAAACACCCCTATTCGTATGATCCGTCCTCATTACTGCCATCCTACTACTCCTCTCCTTACCCGTCCTAGCAGCTGGGATTACCATACTACTTACCGACCGCAACCTTAACACCACATTCTTCGACCCAGCTGGAGGAGGAGACCCAATCCTATACCAACACCT | *Gallus gallus* |
| *Anas platyrhynchos* | TATCTTCGGCGCATGAGCCGGAATAATTGGCACAGCACTCAGCCTACTGATCCGGGCAGAACTAGGCCAGCCAGGGACCCTCCTGGGCGACGACCAAATTTATAACGTGATCGTCACCGCTCACGCCTTCGTAATAATCTTCTTCATGGTAATGCCCATCATAATTGGAGGGTTCGGCAACTGATTGGTCCCCCTGATAATCGGTGCCCCCGACATAGCATTCCCACGAATAAACAACATAAGCTTCTGACTCCTCCCACCATCATTCCTCCTTCTACTCGCCTCATCCACTGTAGAAGCTGGCGCTGGTACGGGTTGAACCGTATACTCACCTCTAGCAGGCAACCTAGCCCACGCCGGAGCCTCAGTGGACCTGGCTATCTTCTCACTTCACCTGGCTGGTGTCTCCTCCATCCTCGGAGCCATTAACTTCATTACCACAGCCATCAACATAAAACCCCCCGCACTCTCACAATACCAAACCCCACTTTTCGTCTGATCAGTCCTAATTACCGCCATCCTGCTCCTCCTATCACTCCCCGTCCTCGCCGCCGGCATCACAATGCTACTAACCGACCGAAACCTAAACACCACATTCTTTGATCCTGCCGGAGGGGGAGACACAATCCTGTACCAACACCTAT | *Anas platyrhynchos* |
| *Anser cygnoides* | ATACCTCATCTTCGGGCCATGAGCAGGAATAGTCGGCACCGCACTCAGCCTATTAATCCGCGCAGAACTAGGACAGCCAGGAACTCTCCTAGGCGACGACCAAATTTACAACGTAATCGTTACCGCCCACGCCTTTGTAATAATCTTCTTTATAGTCATACCCATCATGATCGGAGGATTCGGCAACTGATTAGTCCCCCTCATAATCGGCGCCCCCGACATAGCATTCCCACGAATAAACAACATAAGCTTTTGACTCCTCCCTCCATCATTCCTCCTACTACTAGCCTCATCCACTGTAGAAGCTGGCGCCGGCACAGGCTGAACTGTCTACCCTCCCCTAGCAGGTAACCTCGCCCACGCCGGAGCTTCAGTAGACCTGGCTATCTTCTCACTCCACTTAGCCGGTATCTCCTCCATCCTTGGGGCCATCAACTTTATTACCACAGCTATCAACATAAAACCCCCCGCACTCTCACAATACCAAACCCCACTATTTGTCTGATCCGTACTAATTACCGCCATCCTACTCCTTCTATCACTCCCCGTACTCGCGGCCGGTATTACAATACTACTAACTGATCGAAACCTAAACACCACATTCTTCGATCCGGCTGGAGGGGGAGACCGAATCCTGTACCAACACCT | *Anser cygnoides* |
| *Meleagris gallopavo* | GGCACATGAGCAGGTATAGTCGGCACAGCACTTAGCCTGCTAATCCGTGCAGAACTGGGACAACCTGGGACACTCCTAGGAGACGACCAAATCTATAACGTAATCGTCACAGCCCATGCCTTCGTTATAATCTTCTTTATAGTTATACCTATCATGATCGGAGGCTTCGGTAACTGACTTGTACCACTTATAATTGGTGCCCCAGACATGGCATTCCCACGTATAAATAATATAAGCTTCTGACTCCTTCCACCTTCCTTTCTTCTTCTGCTAGCCTCTTCTACCGTAGAAGCTGGAGCTGGCACTGGATGAACTGTCTACCCACCTTTAGCTAGCAACCTTGCCCACGCTGGTGCATCAGTAGACCTAACTATTTTTTCCCTCCACCTAGCAGGTGTATCCTCCATCCTAGGAGCAATCAACTTTATTACTACTATTATTAACATAAAACCCCCAGCACTGTCACAATACCAAACACCCCTATTTGTTTGATCCGTTCTCATTACCGCTATCCTCCTATTACTCTCTCTACCAGTCCTTGCCGCCGGAATTACAATACTTCTTACTGACCGCAACCTTAACACTACATTCTTTGACCCCGCAGGAGGAGGAGACCCAATCCTATATCAACACC | *Meleagris gallopavo* |
| *Ctenopharyngodon idellus* | TCTTGTATTTGGTGCCTGAGCCGGATAGTGGGAACCGCTCTAAGCCTTCTCATTCGAGCCGAACTAAGCCAACCCGGATCACTTCTGGGCGATGATCAAATTTATAATGTTATTGTCACTGCCCATGCCTTCGTAATAATTTTCTTTATAGTAATACCAATTCTTATTGGAGGGTTTGGAAATTGACTCGTACCATTAATAATTGGAGCACCCGACATAGCATTCCCACGAATAAACAACATGAGTTTCTGACTTCTACCCCCTTCTTTCCTCCTACTATTAGCCTCTTCTGGTGTTGAGGCCGGAGCTGGAACAGGGTGAACAGTTTACCCACCACTCGCAGGCAATCTTGCCCACGCAGGAGCATCCGTAGACCTAACAATTTTCTCACTCCACCTGGCAGGTGTGTCATCAATTTTAGGGGCAATTAATTTTATTACTACAACCATTAACATGAAACCACCAGCCATCTCCCAATACCAAACACCTCTCTTCGTTTGAGCTGTACTTGTAACAGCTGTACTCCTTCTTCTATCTCTACCAGTTCTAGCCGCCGGAATTACAATACTCCTAACAGACCGTAATCTTAACACTACATTCTTTGACCCGGCGGGAGGAGGAGACCCAATTCTTTATCAACACTTAT | *Ctenopharyngodon idella* |
| *Carassius gibelio* | TTTATCTAGTATTTGGTGCCTGAGCCGGATAGTAGGAACCGCTTTAAGCCTCCTCATCCGAGCTGAACTTAGTCAACCCGGATCACTTCTAGGTGATGACCAAATTTACAATGTAATTGTTACCGCCCACGCCTTCGTAATAATTTTCTTTATAGTAATGCCTATCCTCATTGGAGGATTCGGAAACTGACTTGTGCCCCTGATAATCGGAGCCCCAGACATGGCATTCCCACGAATAAATAATATAAGCTTCTGACTTCTTCCCCCATCATTCCTGTTACTACTAGCTTCCTCTGGTGTTGAAGCCGGAGCTGGCACCGGATGGACAGTATACCCCCCTCTTGCAGGAAACCTAGCCCACGCAGGAGCATCAGTAGACCTAACAATTTTCTCACTACATTTAGCAGGTGTTTCATCAATCCTGGGGGCAATCAACTTCATTACTACAACCATTAACATAAAACCTCCAGCCATTTCCCAATACCAAACACCCCTATTTGTTTGATCCGTACTTGTAACCGCCGTCCTCCTTCTCCTATCACTACCTGTTCTAGCTGCCGGTATTACAATGCTTTTAACAGATCGAAATCTTAACACCACATTCTTTGACCCCGCAGGCGGGGGAGACCCAATTCTCTATCAACACTTA | *Carassius gibelio* |
| *Larimichthys crocea* | CTACCTAATTTTTGGTGCATGAGCCGGAATAGTGGGCACAGCCCTAAGTCTCCTAATTCGAGCAGAACTAAGCCAGCCCGGCTCACTTCTCGGAGACGACCAGATTTTTAATGTAATCGTTACGGCACATGCTTTCGTTATAATCTTCTTTATAGTAATACCCGTTATAATTGGAGGGTTCGGGAACTGGCTTGTGCCTTTAATAATTGGCGCCCCCGACATAGCATTCCCCCGAATGAATAACATAAGCTTCTGGCTCATCCCCCCTTCTTTCCTACTGCTCCTCGCCTCATCAGGGGTTGAAGCAGGGGCCGGAACAGGGTGGACAGTCTACCCCCCGCTTGCTGGAAACCTGGCGCACGCAGGGCCTTCAGTCGACTTAGCTATTTTTTCCCTACACCTCGCAGGTGTTTCCTCAATCCTGGGGGCCATCAACTTCATTACAACAATTATTAATATGAAACCCCCCGGCATCACCCAATATCAAACACCTCTGTTTGTCTGAGCCGTTCTAATTACAGCCGTCCTCCTGCTGCTCTCACTACCTGTTTTAGCCGCCGGCATCACAATGCTTTTGACTGACCGCAATCTGAATACAACTTTCTTCGACCCTTCGGGCGGAGGCGATCCCATCCTCTACCAACACCT | *Larimichthys crocea* |
| *Glycine max* | TGCAGAATCCCGTAAACCATCGAGCTTTGAACGCAAGTTGCGCCCGAAGCCATTTAGGCCGAGGGCACGCCTGCCTGGGTGTCACACATCGTTTCCCCAACGCAAACATGTAACAATGTTGTGCGCGGGGTGTATGCTGACCTCGCGCGAGCACGGGCCTCGTGGTTGGTTGAAATCTGGGTTTATGGCCGACTTCGCCGTGATAAAATGGTGGATGAGCCACGCTCGAGACCAATCACGTGCGAGCCGGTCAGTTCTGGACCCATCGACGACCCTTTGTGCACGCACGCTCCCAACGAGACCTCAGGTCAGGCGGGGCTACCCGCTGAGTTTAAGCATATCAATAA | *Glycine max* |
| *Zea mays* | TCGAGTTTTTGAACGCAAGTTGCGCCCGAAGCCTTCTGGCGGAGGGCACGTCTGCCTGGGCGTCACGCCAAAAGACACTCCCAACACCCCCCCGCGGGGCGAGGGACGTGGCGTCTGGCCCCCCGCGCCGCAGGGCGAGGTGGGCCGAAGCAGGGGCTGCCGGCGAACCGCGCCGGGCGCAGCACGTGGTGGGCGACATCAAGTTGTTGTTCTCGGTGCAGCGTCCCGGCGCGCGGCCGGCCATTCGGCCCTAAGGACCCATCGAGCGACCGAGCTTGCCCTCGGACCGCGACCCCAGGTCAGTCGGGACTACCCGCTGAGTTTAAGCATATAAATAAGCGGAGGAGAAGAAACTTACGAGGATTCCCCTAGTAACGGCGAGCGAACCGGGAGCAGCCCAGCT-GAGAATCGCGC | *Zea mays* |

Table S2 Absolute LOD values from real-time PCR for cattle DNA

| Sample species |  | Absolute amount of DNA (ng) | | | | | | | | | |
| --- | --- | --- | --- | --- | --- | --- | --- | --- | --- | --- | --- |
|  | matrix | 1000 | 50 | 10 | 5 | 1.0 | 0.5 | 0.10 | 0.05 | 0.025 | 0.010 |
| *Bos taurus* | donkey | 14.95±0.09 | 19.59±0.11 | 21.96±0.01 | 23.01±0.07 | 25.32±0.04 | 26.96±0.23 | 28.79±0.16 | 29.52±0.13 | 31.05±0.05 | 32.11±0.18 |
|  | sheep |  | 19.61±0.02 | 22.00±0.09 | 23.28±0.20 | 25.38±0.07 | 26.28±0.09 | 28.68±0.29 | 29.53±0.09 | 30.86±0.12 | 31.10±0.10 |
|  | pork |  | 18.76±0.03 | 21.33±0.05 | 22.39±0.05 | 24.60±0.07 | 25.58±0.02 | 27.81±0.14 | 28.86±0.05 | 30.05±0.06 | 31.87±0.31 |
|  | horse |  | 19.52±0.02 | 21.02±0.03 | 22.73±0.15 | 25.23±0.07 | 26.22±0.04 | 28.39±0.36 | 29.98±0.32 | 30.32±0.64 | 31.83±0.10 |
|  | rabbit |  | 19.11±0.09 | 21.44±0.06 | 22.4±0.09 | 24.67±0.04 | 25.84±0.01 | 26.72±0.15 | 29.04±0.12 | 30.15±0.04 | 31.74±0.44 |
|  | chicken |  | 19.61±0.09 | 22.14±0.05 | 22.95±0.09 | 25.27±0.01 | 26.38±0.02 | 28.42±0.15 | 29.54±0.08 | 29.68±0.07 | 30.98±0.09 |
|  | duck |  | 19.46±0.02 | 21.83±0.02 | 23.19±0.30 | 25.12±0.15 | 26.28±0.10 | 28.43±0.20 | 29.66±0.01 | 30.77±0.18 | 31.79±0.14 |
|  | goose |  | 19.48±0.03 | 21.88±0.03 | 22.85±0.01 | 25.25±0.05 | 26.91±0.29 | 28.34±0.10 | 29.42±0.07 | 30.50±0.08 | 31.39±0.26 |
|  | grass carp |  | 19.42±0.02 | 21.95±0.04 | 22.99±0.07 | 25.48±0.02 | 26.39±0.01 | 28.59±0.18 | 29.63±0.13 | 30.70±0.16 | 31.87±0.19 |
|  | soybean |  | 19.57±0.01 | 21.93±0.13 | 23.08±0.05 | 25.33±0.01 | 26.32±0.12 | 28.51±0.12 | 29.61±0.03 | 29.78±0.08 | 31.18±0.07 |
|  | corn |  | 19.84±0.12 | 22.02±0.05 | 21.82±0.14 | 25.35±0.07 | 26.45±0.08 | 28.35±0.25 | 29.50±0.03 | 31.23±0.15 | 32.31±0.51 |

In this work, negative amplification control (NAC) were set up, and it is no amplification signal.

Extraction DNA concentration from all samples were within the 10^2^ range.

The Cq value of less than 32 is considered to detect bovine ingredient.

Table S3 Relative LOD values from real-time PCR for cattle DNA

|  | | | Percentage (w/w) | | | | | | | | | |
| --- | --- | --- | --- | --- | --- | --- | --- | --- | --- | --- | --- | --- |
| Sample species | matrix | code | 100% | 5% | 1% | 0.5 | 0.1 | 0.05 | 0.01 | 0.005 | 0.002 | 0.001 |
|  | donkey | Aa1-9^d^ | 14.45±0.06 | 17.20±0.08 | 19.18±0.66 | 20.49±0.03 | 22.25±0.05 | 23.12±0.11 | 25.50±0.05 | 26.89±0.11 | 27.67±0.17 | 32.87±0.37 |
| *Bos taurus* (raw) | sheep | Ab1-9 |  | 20.69±0.13 | 21.84±0.03 | 23.90±0.08 | 24.73±0.06 | 26.08±0.18 | 29.18±0.06 | 30.31±0.06 | 31.59±0.69 | 34.42±0.60 |
|  | pork | Ac1-9 |  | 19.68±0.08 | 21.3±0.02 | 21.57±0.07 | 23.97±0.10 | 25.05±0.16 | 28.10±0.10 | 29.19±0.16 | 30.66±0.24 | —— |
|  | soybean | Ad1-9 |  | 18.01±0.07 | 20.71±0.19 | 23.37±0.39 | 23.56±0.03 | 25.93±0.05 | 26.94±0.08 | 28.17±0.05 | 29.62±0.10 | 33.50±2.21 |
|  | donkey | Aa1-9 |  | 22.71±0.04 | 22.82±0.01 | 23.68±0.48 | 24.85±0.08 | 26.17±0.78 | 26.08±0.03 | 26.48±0.03 | 27.74±0.33 | 31.58±0.89 |
| *Bos taurus* (autoclaved) | sheep | Ab1-9 |  | 16.77±0.18 | 18.01±1.11 | 20.11±0.29 | 26.25±0.07 | 25.67±0.22 | 26.35±0.62 | 27.74±0.22 | 29.23±0.18 | —— |
|  | pork | Ac1-9 |  | 19.03±0.12 | 21.83±0.07 | 22.23±0.03 | 23.45±0.01 | 24.32±0.12 | 27.53±0.10 | 28.31±0.12 | 29.63±0.19 | —— |
|  | soybean | Ad1-9 |  | 16.60±0.41 | 18.84±0.19 | 22.12±0.18 | 22.20±0.20 | 24.53±0.16 | 27.36±0.49 | 28.61±0.16 | 29.80±0.16 | —— |

In this work, negative amplification control (NAC) were set up, and it is no amplification signal.

Extraction DNA concentration from all samples were within the 10^2^ range.

The Cq value of less than 32 is considered to detect bovine ingredient.

**Fig. S1.** Normalized calibration curve for the reference mixture with the average Cq values of reference mixtures using the endogenous PCR system (n=8,8)

**Fig. S2.** Normalized calibration curve for the reference mixture with the average Cq values of reference mixtures using the endogenous PCR system (n=5, 7)

**Fig. S3.** Normalized calibration curve for the reference mixture with the average Cq values of reference mixtures using the endogenous PCR system (n=8, 5)

**Fig. S1**


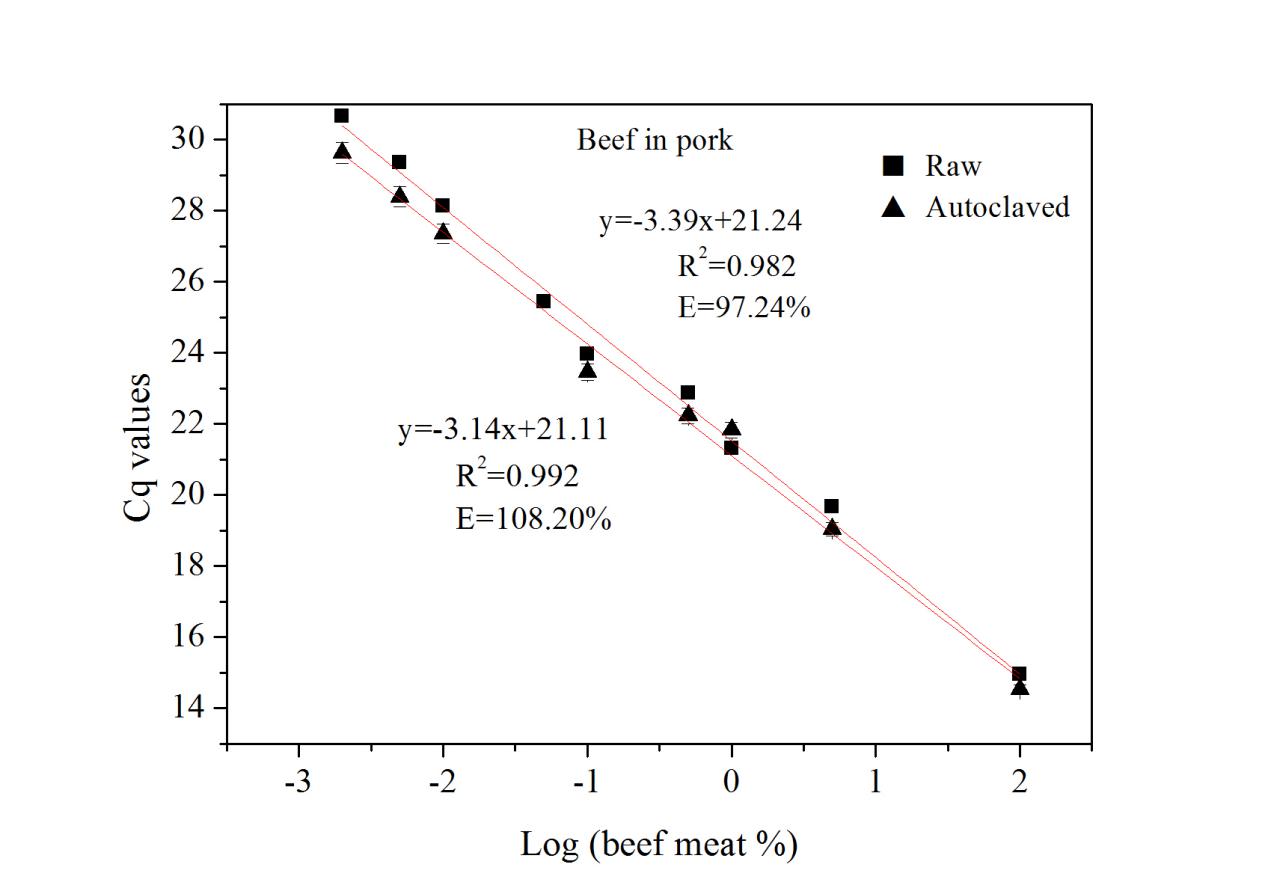


**Fig. S2**


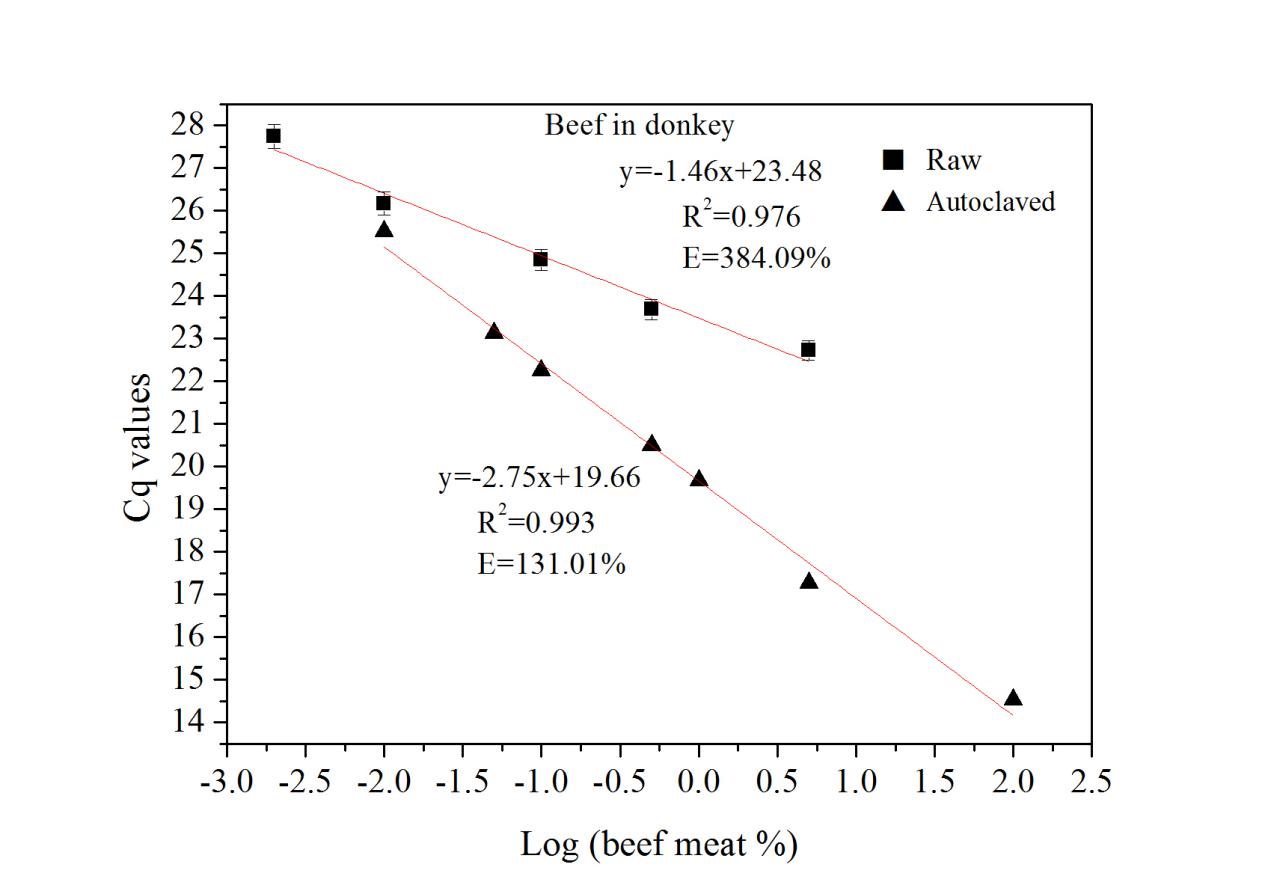


**Fig. S3**


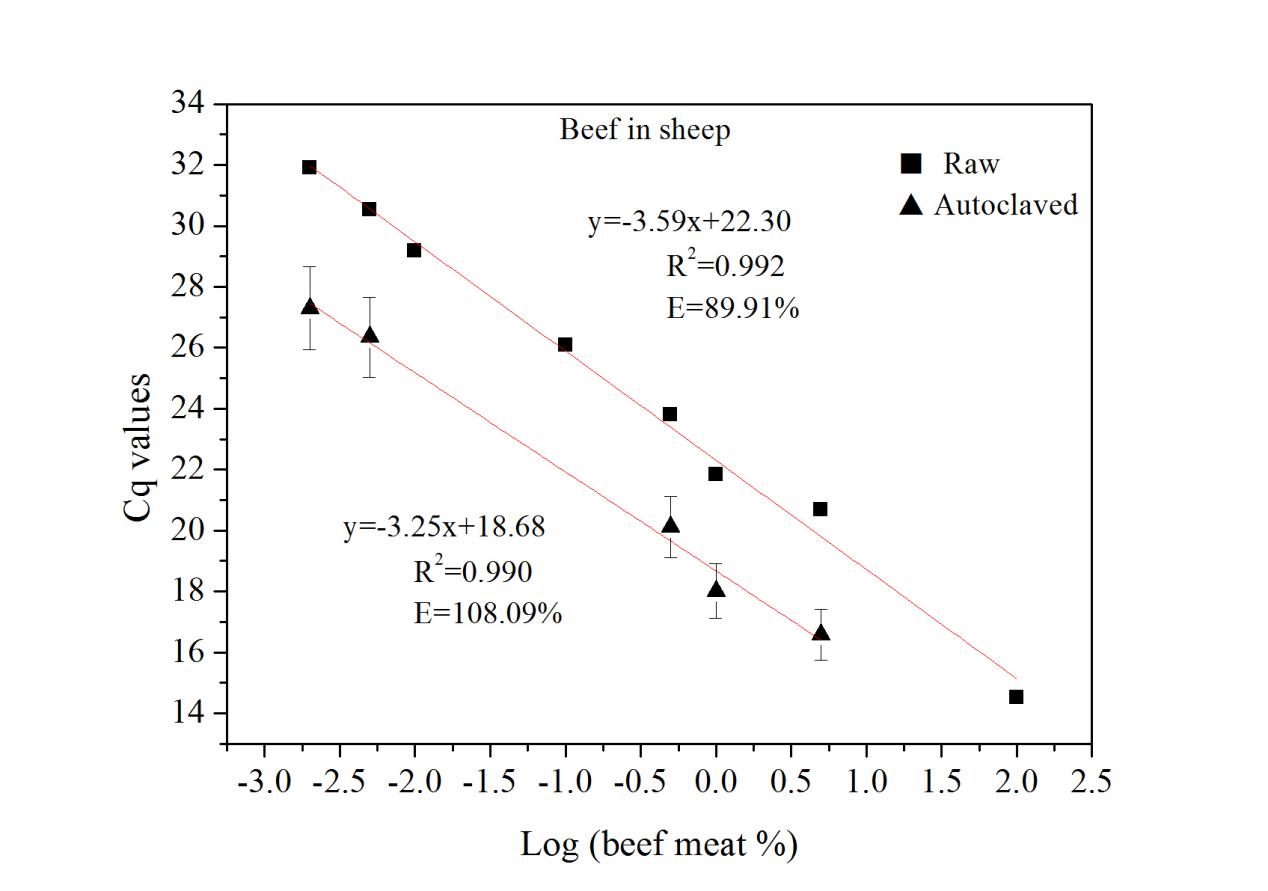

Supplement: Supplementary file 1 — Supplementary information [file 41598_2020_59010_MOESM1_ESM.doc]
